# Outcome of uncomplicated ureteric calculi managed with medical expulsive therapy in the outpatient clinic of a urology unit in Sri Lanka

**DOI:** 10.1186/s13104-017-2974-1

**Published:** 2017-11-28

**Authors:** Malaka Dharmakeerthi Jayawardene, Balasingam Balagobi, A. L. A. M. C. Ambegoda, Sanjeewa Vidanapathirana, G. W. A. S. K. Wijayagunawardane, V. Senthan, D. D. Ranasinghe, Anuruddha M. Abeygunasekera

**Affiliations:** 1 Post Graduate Institute of Medicine, University of Colombo, Colombo, Sri Lanka; 2 Department of Urology, Colombo South Teaching Hospital, Kalubowila, Sri Lanka; 3 Department of Radiology, Colombo South Teaching Hospital, Kalubowila, Sri Lanka

**Keywords:** Ureteric calculi, Sri Lanka, Medical expulsive therapy, α Blockers, Tamsulosin

## Abstract

**Objective:**

Although medical expulsive therapy (MET) is shown to be effective for ureteric calculi, the optimum duration and the stone size suitable for MET are not well established yet. The objectives of the study were to determine the optimum duration and maximum stone size suitable for MET.

**Results:**

All patients with radiologically confirmed uncomplicated ureteric calculi treated with MET using tamsulosin over a period of 6 months in the outpatient setting were followed up. There were 213 patients. 165 were men. Mean age was 42 years. At presentation 42 stones were in upper ureter (19.7%), 51 in mid ureter (23.9%), 120 in lower ureter (56.3%). The majority (82.7%) of stones were less than 10 mm. Seven stones (3.3%) were over 15 mm. Ninety-two (43.2%) patients had spontaneous passage of stones within 6-weeks of MET. Another 38.9% passed the stone within the next 6-weeks. Thirty-eight patients (17.8%) required surgery. Uncomplicated ureteric stones up to 10 mm can be given a trial of MET using tamsulosin which can be extended up to 12-weeks with a success rate over 92%. This may have substantial clinical and fiscal benefits by reducing the number of interventional procedures especially in resource-poor settings.

**Electronic supplementary material:**

The online version of this article (10.1186/s13104-017-2974-1) contains supplementary material, which is available to authorized users.

## Introduction

Urolithiasis has a prevalence of around 10% in adults [1]. About 9% of urolithiasis related deaths in the UK are due to ureteric stones [2]. With the advancement of technology related to shock wave lithotripsy (SWL) and ureteroscopic lasertripsy, there is a tendency to offer interventions to remove ureteric stones more frequently [3].

Expectant management of ureteric stones is popular among patients in Sri Lanka and this is evident by the fact that most patients with ureteric colic try native treatments to promote spontaneous passage. Furthermore surgical interventions can be risky and costly [4]. The potential complications include sepsis, stent morbidity and ureteric damage. The average cost of uretroscopic lasertripsy in fee-levying hospitals of Sri Lanka is around SLR 200,000/= (1500 US$). Inappropriate care is a widespread phenomenon. Medical personnel in both high income countries and low-middle income countries overuse ineffective but familiar, lucrative or otherwise convenient services, despite marginal benefits to patients [5]. Unnecessary and avoidable surgery in health systems which have waiting lists for surgery has a negative impact on patients who really deserve and benefit from surgery [6].

Traditionally the expectant management is reserved only for renal stones smaller than 5 mm [7]. Recent studies have shown that medical expulsive therapy (MET) is efficacious in promoting stone expulsion and this effect is more prominent with larger stones [8, 9]. Tamsulosin and nifedipine are the two main agents shown to be effective as MET [10]. The ureter contains α adrenergic receptors along its entire length with the highest concentration in the distal ureter [11, 12]. Tamsulosin, a selective α1A adrenergic receptor antagonist reduces spasms in the ureter allowing urine to be accumulated above the stone that results in an increase in the intra-ureteral pressure above the stone, while decreasing peristalsis in the ureter distal to the stone which reduces intra-ureteral pressure below the stone. This pressure gradient is thought to increase the fluid bolus volume transported down the ureter, promoting painless expulsion of the stone. Nifedipine reduces ureteral spasms by blocking calcium channels promoting stone passage [13–15]. The optimum duration of MET before abandoning it and the upper limit of the stone size where MET can be tried are not well established yet. One meta-analysis also raises the possibility of international differences in baseline rates of stone passage and suggests research studies in different ethnicities [8].

Sri Lanka is an island nation in South Asia with a population of 21 million. It is a tropical country with a weather temperature ranging between 27–31 °C and categorised as a low-middle income country. Composition patterns of Sri Lankan urinary calculi are shown to be different from the West [16]. There are no published data available on the efficacy of MET in ureteric stones among Sri Lankan patients. Hence we decided to study the outcome of patients with uncomplicated ureteric calculi encountered in the outpatient setting of Sri Lanka.

## Main text

### Methods

This was a prospective study conducted in a urology unit of a Teaching Hospital in Sri Lanka. A convenience sample consisting of all adult patients with newly diagnosed and radiologically confirmed ureteric calculi who attended the outpatient clinic from 1 October 2016 to 31 March 2017, formed the study population. Data related to demography, stone size, stone location, radiological investigations and the outcome were recorded prospectively.

All patients with clinical suspicion of a ureteric stone had a plain X-ray of kidneys, ureters, and bladder (KUB) and ultrasound scan (USS) of KUB. When the combination of X-ray and USS could not demonstrate a stone, but the clinical suspicion was high, a non-contrast computerized tomography (NCCT) of KUB was done. Tamsulosin which has been proven to be useful as an effective drug for MET was the medication used in this study. Once the diagnosis was made, patients were prescribed tamsulosin 0.4 mg daily for 6 weeks initially. Non-steroidal anti inflammatory drugs (NSAIDs) in the form of diclofenac sodium and celecoxib were prescribed along with omeprazole or rabeprazole for pain relief. The duration of NSAIDS was 2 weeks, but was modified according to the severity of pain. Patients with stone size above 10 mm were enlisted for surgery and a scheduled date was given—ureteroscopic lasertripsy for stone size of 11–15 mm and open ureterolithotomy for over 15 mm. However, as there was a waiting time for such surgery at Colombo South Teaching Hospital those patients were also prescribed MET and followed up until the time of surgery.

Patients with evidence of urosepsis, renal impairment, pregnancy, breast feeding and a single functioning kidney were excluded from the study. Those with fever, high C-reactive protein (CRP) level (more than 3 mg/L) or ultrasonographic evidence of pyelonephritis were considered as having urosepsis. Renal impairment was diagnosed when serum creatinine was elevated than the highest normal value of the laboratory of the institute (more than 130 µmol/L).

The stone size measured was the maximum diameter in millimeters by USS, digitally enhanced X-ray or NCCT. For inferential statistical purposes the stone sizes were segregated into four groups (< 5 mm, 5–10 mm, 11–15 mm, > 15 mm).

The outcome of MET was evaluated at the end of 6- and 12-weeks. Stone passage was defined as absence of symptoms and normal radiological investigations which were positive at the beginning of the study. When the conclusion was in doubt, a non-contrast CT scan of urinary tract (NCCT KUB) was done to confirm the absence of calculi. Those who did not expel the stone by 6-weeks were continued on tamsulosin for another 6-weeks but were included in the waiting list for ureteroscopic lasertripsy using a Ho:YAG laser or open ureterolithotomy.

Chi squared test and correlation coefficient were used for the statistical analysis and p < 0.05 was considered statistically significant. All study participants gave informed, written consent to be included in the study. Approval for the study was obtained from the Ethics Review Committee of the Institute.

### Results

There were 238 patients with radiologically confirmed ureteric calculi during the study period. Twenty-five did not complete the follow up and were excluded from the study. Hence 213 patients were included in the final analysis. There were 165 (77.4%) men with a male to female ratio of 3.4:1. The mean age was 42 years (range 17–83). Most (47.8%) belonged to the age group of 20–40 years. Sixty-two (29.1%) patients gave a history of taking treatment for a urinary stone in the past. Family history of urolithiasis was evident in 20 (9%).

Stone characteristics are summarized in Table 1. Majority of the stones were in the lower ureter (56.3%). Only 19.7% of stones were found in the upper ureter at the time of presentation. While 31.5% of stones were less than 5 mm in size, the majority (51.2%) were between 6 and 10 mm. Seven (3.3%) patients had stones larger than 15 mm. The ureteric stone was identified in 209 (98.1%) patients using the combination of USS and X-ray KUB. Only four patients required NCCT KUB for stone detection.

**Table 1:** Stone characteristics and outcome (n = 213)

Characteristic	n	%
Location		
Upper ureter	42	19.7
Mid ureter	51	23.9
Lower ureter	120	56.3
Stone size		
< 5 mm	67	31.5
5–10 mm	109	51.2
11–15 mm	30	14.1
> 15 mm	7	3.3

Outcome	N	%
Stone passed with in 6 weeks	92	43.2
Stones passed with in 6–12 weeks	83	38.9
Surgery required	38	17.8
Total	213	100

All 213 patients were started on MET at the beginning of the study irrespective of the stone size. However 30 patients with stones between 11 and 15 mm were scheduled for ureteroscopic lasertripsy too. Seven patients with stones larger than 15 mm were scheduled for open ureterolithotomy. SWL was not available at the institute. At the end of 6-weeks, 92 (43.2%) patients had passed the stone (Table 1). Another 83 (38.9%) passed the stone within the next six-weeks. All patients with stones less than 5 mm passed the stone with MET by 12 weeks. Ninety-seven patients with stones between 6 and 10 mm passed the stone with MET. Eight patients with stones between 11 and 15 mm (29.6%) passed the stone but 19 (70.3%) such patients required surgical intervention. Eighteen of them had ureteroscopic lasertripsy. In two patients the stone migrated to the lower ureteric orifice with MET enabling ureteric meatotomy and extraction of the stone. All patients with stones larger than 15 mm required surgery though there was some downward movement of the stones with MET. Finally 38 (17.8%) patients required surgical intervention.

While all stones less than 5 mm spontaneously passed, 90% of the stones sized between 6 and 10 mm also passed with MET within 12-weeks (Table 2). The success of MET significantly improved when the distance to travel along the ureter for the stone was less. Only 47.6% of upper ureteric stones had spontaneous expulsion with MET, though 82.3% of mid ureteric stones and 94.1% of lower ureteric stones passed with MET. While 52.4% of upper ureteric stones required surgery, only 6% of lower ureteric stones required surgery (Table 2).

**Table 2:** Success after 12 weeks of medical expulsive therapy (MET)

Stone characteristic	MET n	%	Surgery n	%
Location				
Upper ureter	20	47.6	22	52.4
Mid ureter	42	82.3	9	17.7
Lower ureter	113	94.1	7	5.9
Total	175	82.1	38	17.9
Stone size				
< 5 mm	67	100	0	0
5–10 mm	98	90	11	10
11–15 mm	10	33	20	67
> 15 mm	0	0	7	100
Total	175	82.1	38	17.9

**Fig. 1:**
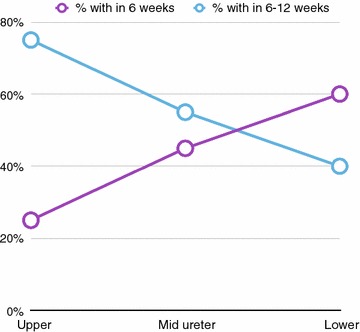
Association between the time of passage and site of the stone

We also analysed the relationship between the time of passage of the stone and the stone size and location. Lower ureteric stones constituted 74% of the stones passed within the first 6-weeks, while upper ureteric stones constituted only 5%. In comparison, out of the stones that passed subsequently between 6 and 12 weeks, this percentage occupied by the lower ureteric stones dropped to 54% while the percentage of stones in the upper ureter that passed with MET increased to 18%. Out of the upper ureteric stones that passed in the 6-12 week period, 93.3% of stones were less than 10 mm. This statistically significant negative correlation between the time and site of the stone was conspicuous when the stones that passed from the three locations of the ureter were separately considered over time (Fig. 1). Stones that passed from upper ureteric level had a three-fold rise in stone passage during the second 6-weeks (25% passed in first 6-weeks, while remaining 75% passed in the next 6-weeks). This pattern was reversed for lower ureteric stones (60% passing the stone in the first 6-weeks and remaining 40% in the second 6-weeks) (Fig. 1).

Analysis of data in relation to demographic parameters and patient risk factors for stone formation revealed that the success rate of MET did not depend on the gender (p = 0.851), age (p = 0.92), past history of urolithiasis (p = 0.611), or family history of urolithiasis (p = 0.774).

### Discussion

According to the established evidence, 95% of ureteric stones up to 4 mm would spontaneously pass without any medical or surgical intervention within 40-days [17]. Stones less than 5 mm in our study also showed a similar expulsion rate. In addition, our results show that 48.8% of ureteric stones up to 10 mm pass within 6-weeks of MET and 93.2% by 12-weeks. Furthermore our study shows a statistically significant correlation between the size of the stone, site of the stone and success rate of medical expulsion therapy. The linear relationship between the site of the stone and the success rate of MET was only evident in stones less than 10 mm in size (see Additional file 1: Figures S1, S2). Therefore it is reasonable to propose that uncomplicated ureteric stones less than 10 mm should be managed with MET initially. The trial period of MET can be extended up to 12-weeks if the pain is well controlled and there is clinical and radiological evidence of downward migration of the stone. However such patients need careful follow up so that the minority of those who do not get relief of symptoms or stone expulsion should be offered surgical intervention. About 25% of patients with ureteric calculi undergo urological procedures in the developed world compared to 17.8% in our study cohort [18]. Most patients had mild to moderate hydronephrosis but with passage of stones this was reversed and none of the patients had biochemically evident deterioration of renal functions.

The biggest strength of this observational study is that it was done in the routine care setting. This helps to extrapolate the findings directly to clinical practice and induce necessary changes in existing practice. This type of pragmatic observational studies would allow better clinical trial planning and promote equity of health care services among populations of different socioeconomic strata [6]. Even developed countries can benefit as these results contribute to reverse innovation [19].

### Conclusions

Only a minority of patients with ureteric calculi require surgical intervention. Uncomplicated ureteric stones up to 10 mm can be given a trial of MET initially. The trial period may be extended up to 12-weeks depending on the control of symptoms and downward movement of the stone.

#### Limitations

There are few limitations in our study. CT scanning was not used to assess the size of the stone which would have been more precise. The adverse effects of drug treatment were not evaluated in a comprehensive manner. Yet the findings of our study will be of relevance to practicing clinicians in this evidence-poor area in urology due to its pragmatic nature.

## Additional file

**Additional file 1: Figure S1.** Spontaneous passage vs surgery in stones < 10 mm in relation to site. **Figure S2.** Spontaneous passage vs surgery in stones > 10 mm in relation to site.

## Abbreviations

MET: medical expulsive therapy
SWL: shockwave lithotripsy
SLR: Sri Lankan rupees
KUB: kidney, ureter, bladder
USS: ultrasound scan
NCCT: non-contrast computerized tomography
NSAIDs: non-steroidal anti inflammatory drugs
CRP: C-reactive protein
