# Supplementary material for: Outcome of uncomplicated ureteric calculi managed with medical expulsive therapy in the outpatient clinic of a urology unit in Sri Lanka
Source: BMC Res Notes. 2017 Nov 28;10:636. doi: 10.1186/s13104-017-2974-1 (PMC5704535; doi:10.1186/s13104-017-2974-1)
Supplement: Supplementary file 1 — Additional file 1: Figure S1. Spontaneous passage vs surgery in stones < 10 mm in relation to site. Figure S2. Spontaneous passage vs surgery in stones > 10 mm in relation to site. [file 13104_2017_2974_MOESM1_ESM.pdf]

Additional file 1:

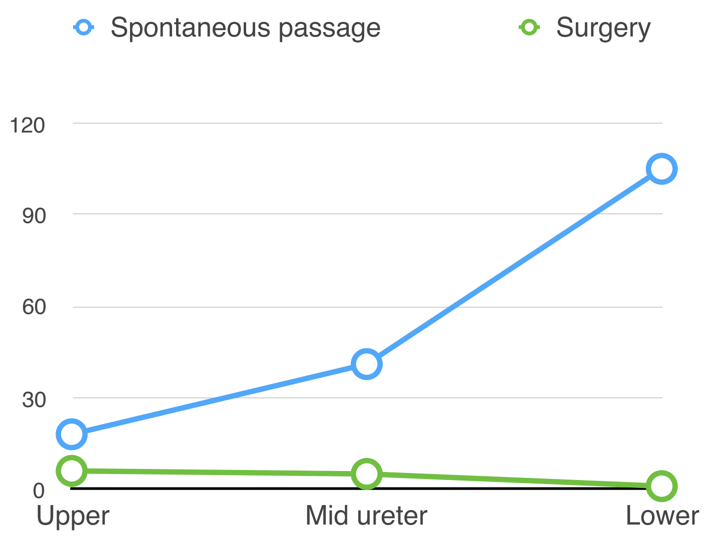

Figure S1. Spontaneous passage vs surgery in stones < 10 mm in relation to site

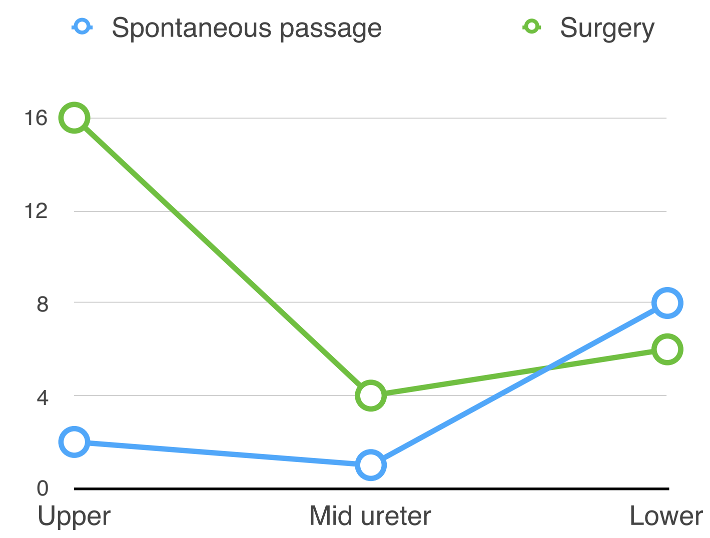

Figure S2. Spontaneous passage vs surgery in stones > 10 mm in relation to site
